# Supplementary material for: FastqPuri: high-performance preprocessing of RNA-seq data
Source: BMC Bioinformatics. 2019 May 3;20:226. doi: 10.1186/s12859-019-2799-0 (PMC6500068; doi:10.1186/s12859-019-2799-0)
Supplement: Supplementary file 2 — Archive of FastqPuri. Archive containing all files needed to install and run FastqPuri v1.0.6. Date stamp March 22, 2019. (GZ 47,819 kb) [file 12859_2019_2799_MOESM2_ESM.gz › FastqPuri-1.0.6/html/globals_g.html]

FastqPuri: File Members


FastqPuri


Here is a list of all documented file members with links to the documentation:

### - g -

- get\_first\_tile()
  : stats\_info.c
  , stats\_info.h
- get\_fqread()
  : fq\_read.h
  , fq\_read.c
- get\_new\_pool()
  : tree.c
  , tree.h
- get\_tile\_lane()
  : stats\_info.c
- getarg\_makeBloom()
  : init\_makeBloom.c
  , init\_makeBloom.h
- getarg\_makeTree()
  : init\_makeTree.c
  , init\_makeTree.h
- getarg\_Qreport()
  : init\_Qreport.c
  , init\_Qreport.h
- getarg\_Sreport()
  : init\_Sreport.h
  , init\_Sreport.c
- getarg\_trimFilter()
  : init\_trimFilter.c
  , init\_trimFilter.h
- getarg\_trimFilterDS()
  : init\_trimFilterDS.h
  , init\_trimFilterDS.c
- GLOBAL
  : defines.h
- GOOD
  : defines.h
- GOOD2
  : defines.h


---

Generated on Mon Mar 19 2018 23:42:01 for FastqPuri by  

 1.8.14
